# Paediatric glaucoma in Scotland

**DOI:** 10.1186/s12886-020-01347-7

**Published:** 2020-02-27

**Authors:** Daniel Beck, Martin Galea, Cheng Yi Loo, Conrad Schmoll, Frederick R. Burgess, Donald Montgomery, Andrew J. Tatham

**Affiliations:** 1 Princess Alexandra Eye Pavilion, Chalmers Street, Edinburgh, EH3 9HA UK; 2 Tennent Institute of Ophthalmology, Gartnavel General Hospital, 1053, Great Western Road, Glasgow, G12 0YN UK; 3 University of St Andrews School of Medicine, North Haugh, St Andrews, Fife, KY16 9TF UK

**Keywords:** Paediatric glaucoma, Paediatric ophthalmology, Glaucoma surgery, Service planning, Scotland

## Abstract

**Background:**

The primary aim was to estimate the incidence of primary and secondary childhood glaucoma in Scotland over a 2-year period. The secondary aim was to gauge the confidence and experience of ophthalmologists in Scotland in managing these patients.

**Methods:**

A 7 question electronic survey was distributed to all consultant members of the Scottish Paediatric Club and Scottish Glaucoma Club. Respondents were asked to report the number of cases and types of childhood glaucoma they had managed in the last 2 years. Respondents were also asked about experience and confidence in a range of glaucoma procedures, number of patients requiring referral to specialist centres and interest in the development of a centre of excellence in Scotland.

**Results:**

The survey returned a 56% response rate, reporting 85 new cases of paediatric glaucoma in Scotland over the preceding 2 years. 11 (12.9%) had primary glaucoma and 74 (87.1%) had secondary glaucoma. The most common subtype of secondary glaucoma was uveitic glaucoma (*n* = 29). None of the respondents declared confidence or experience in trabeculotomy or goniotomy procedures. Eleven children required referral to a specialist unit outside Scotland. 85.7% of respondents felt Scotland would benefit from a specialist unit for paediatric glaucoma.

**Conclusions:**

This survey reflects an appetite for a specialist service for paediatric glaucoma in Scotland. However, further consideration is needed to determine if there is sufficient patient load to maintain such a service.

## Background

Childhood glaucoma is a rare and potentially blinding condition responsible for 5% of childhood blindness worldwide [1, 2]. It is estimated to account for 1.2% of blindness in children in the United Kingdom but up to 7% of childhood blindness in Southern India, the region with the highest reported rate [3].

The Childhood Glaucoma Research Network (CGRN) recently proposed a novel classification system for childhood glaucoma which was agreed at the 9th World Glaucoma Association Consensus meeting (Beck A, et al. Section 1: Definition, classification, differential diagnosis. In: Weinreb, RN, et al., editors World Glaucoma Association Consensus Series 9: Childhood Glaucoma. Amsterdam, Netherlands: Kugler Publications 2013. pp. 3–10). The CGRN classification system describes 6 types of childhood glaucoma; 1) glaucoma following cataract surgery, 2) glaucoma associated with non-acquired systemic disease or syndrome (e.g. phacomatoses, connective tissue disorders), 3) glaucoma associated with non-acquired ocular anomalies (e.g. Peter’s anomaly, Axenfeld-Rieger anomalies), 4) glaucoma associated with acquired conditions (e.g. glaucoma associated with retinopathy of prematurity, steroids or uveitis), 5) primary congenital glaucoma (PCG) and 6) juvenile open angle glaucoma. PCG, which is characterised by buphthalmos in addition to two or more of raised intraocular pressure (IOP), optic nerve cupping, Haab striae, visual field defect or abnormal increase in axial length, is subclassified as neonatal onset (≤1 month of age at onset), infantile onset (> 1 to 24 months at onset) and late onset (> 2 years of age at onset).

Childhood glaucoma is rare, with PCG the most common type [3]. The British Infantile and Childhood Glaucoma (BIG) Eye Study found that, in the UK, the annual incidence of diagnosis of PCG was 5.41 in 100,000 (1/18,500) live births [4]. This study also found that ethnic minorities from South Asia are at significantly increased risk of PCG, with children of Pakistani origin having almost 9-times the incidence of PCG compared to Caucasians. Similarly, the incidence of PCG has been found to be higher in India and the Middle East [3]. The BIG Eye study included children with PCG from Scotland. Six cases were reported in Scotland compared to twenty-nine in England and two in The Republic of Ireland. Taking population into account, Scotland had twice the annual incidence of PCG compared to England, although the reason for this was uncertain [4].

The prognosis of paediatric glaucoma is highly dependent on early accurate diagnosis and appropriate management with good IOP control It can easily be missed in the early stages, leading to potentially irreversible corneal and optic nerve damage [4, 5]. It is estimated that an ophthalmologist in a non-specialist centre in the western world will only see a new case of PCG approximately every 5 years [4, 5]. This rarity creates challenges regarding treatment. In addition, many children with glaucoma require glaucoma surgery, but this is technically challenging and requires an experienced surgeon. Given that childhood glaucoma is rare, it may be difficult for surgeons to treat sufficient numbers of children to gain and maintain competence hence, together with other rare diseases, there has been a trend towards managing childhood glaucoma in large supra-regional referral centres.

The aim of this study was to estimate the incidence of childhood glaucoma in Scotland and to gauge the confidence and experience of ophthalmologists in managing these patients. We also sought to determine patterns of referral to specialist centres and to establish whether Scotland, a country of 5 million people, might benefit from a specialist paediatric glaucoma unit.

## Methods

An electronic questionnaire was distributed to all 50 consultant members of the Scottish Glaucoma Club and Scottish Paediatric Ophthalmology Club using the REDCap (Research Electronic Data Capture) electronic data capture tool. REDCap is a secure web-based application designed to support data capture for research studies [6]. Members of both societies were identified from email lists held by society secretaries. The initial questionnaire was emailed to all members in May 2016.

**Fig. 1:**
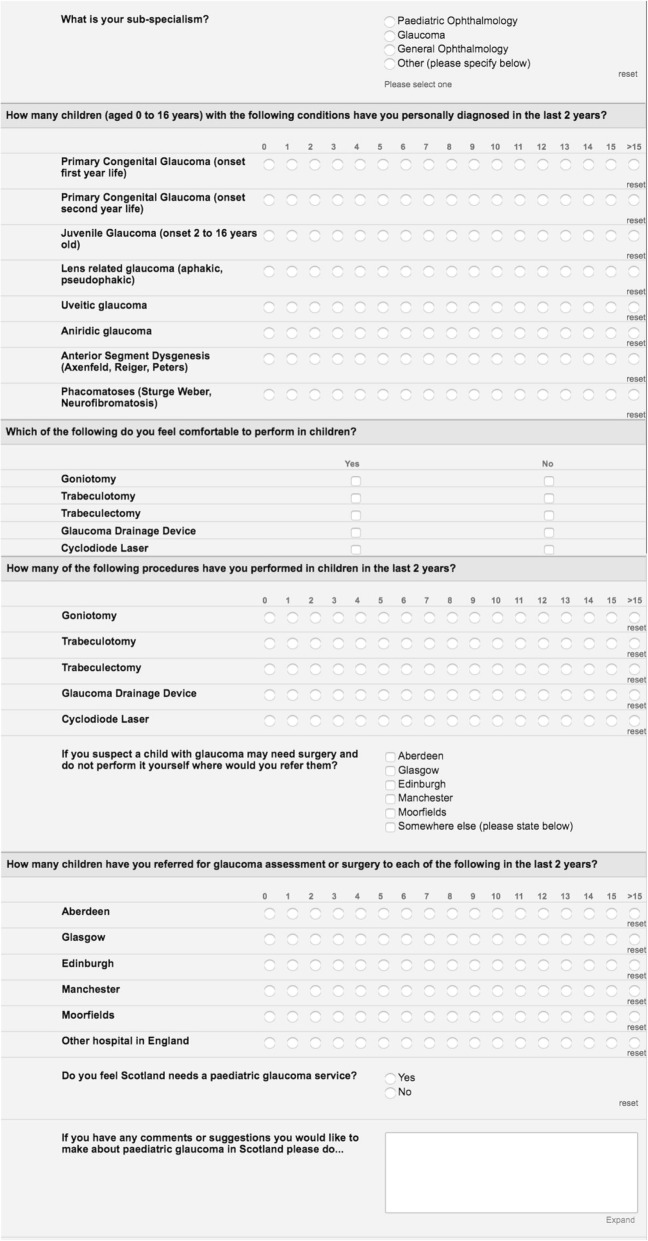
Screenshot showing the electronic survey questions and possible responses sent to all participants

The survey consisted of 7 questions shown in Fig. 1. Questions included; what is your sub-speciality? How many children (aged 0 to 16) have you personally diagnosed with various types of childhood glaucoma in the last 2 years? Options included primary congenital glaucoma, juvenile glaucoma, glaucoma following cataract surgery, glaucoma associated with acquired conditions e.g. uveitic glaucoma, glaucoma associated with non-acquired systemic disease or syndromes, e.g. phacomatoses, and glaucoma associated with non-acquired ocular anomalies e.g. aniridic glaucoma, anterior segment dysgenesis. Other questions enquired as to which glaucoma procedures the responder felt comfortable performing in children, with specific questions enquiring about goniotomy, trabeculotomy, trabeculectomy, glaucoma drainage device surgery and transcleral cyclodiode laser. We also questioned how many of each of these procedures clinicians had performed in children in the last 2 years. The next questions related to where the responder would refer a child with glaucoma who may need surgery and how many children the responder had referred in the last 2 years. Finally, we asked whether the responder felt that Scotland needs a paediatric glaucoma service and invited any comments.

Questions regarding respondents’ identity or home institution were deliberately avoided. This allowed responders to be as open as possible in their answers. The questionnaire was kept open for a period of 3 months and two reminder emails were sent to non-responders.

## Results

Twenty-eight out of 50 consultant ophthalmologists (56%) responded to the survey. Of those responding, 11 (39.3%) reported a subspecialist interest in paediatric ophthalmology, 11 (39.3%) were glaucoma specialists, and 2 (7.1%) general ophthalmologists. Four responders (14.3%) did not disclose their sub-specialist interest.

In total there were 85 cases of childhood glaucoma reported over the two-year period. The most common form of childhood glaucoma was glaucoma associated with acquired conditions, primarily uveitis. Eight responders (28.6%) stated they had diagnosed at least one case of uveitic glaucoma (6 reporting 1 case, 1 reporting 2 cases, 1 reporting 3 cases and 2 reporting 10 cases diagnosed), for a total of 29 children with uveitic glaucoma alone. Next most common was glaucoma following cataract surgery which was encountered by 8 of 28 responders (28.6%). Six reported personally diagnosing 1 case, 1 reported diagnosing 4 patients and 1 reported diagnosing more than 15 cases in the preceding 2 years, for a total of at least 25 children with a new diagnosis of glaucoma following cataract surgery detected. There were 6 cases of PCG (neonatal or infantile) reported by 6 responders, indicating 21.4% of responders had personally diagnosed one case of PCG in the last 2 years. There were 5 cases of juvenile open angle glaucoma, reported by 4 responders. Glaucoma associated with non-acquired systemic disease or syndromes and glaucoma associated with non-acquired ocular anomalies were far less common but 4 cases of aniridic glaucoma, and 8 cases of glaucoma associated with anterior segment dysgenesis and 8 associated with phacomatoses were reported (Table 1).

**Table 1:** Number of children diagnosed with glaucoma, by subtype, in Scotland over a 2 year period

Primary Glaucoma (*n* = 11)	Secondary Glaucoma (*n* = 74)
PCG (*n* = 6)	Lens related (*n* = 25)
JOAG (*n* = 5)	Uveitic (*n* = 29)
	Aniridic (*n* = 4)
	Anterior segment dysgenesis (*n* = 8)
	Phacomatosis (*n* = 8)

**Fig. 2:**
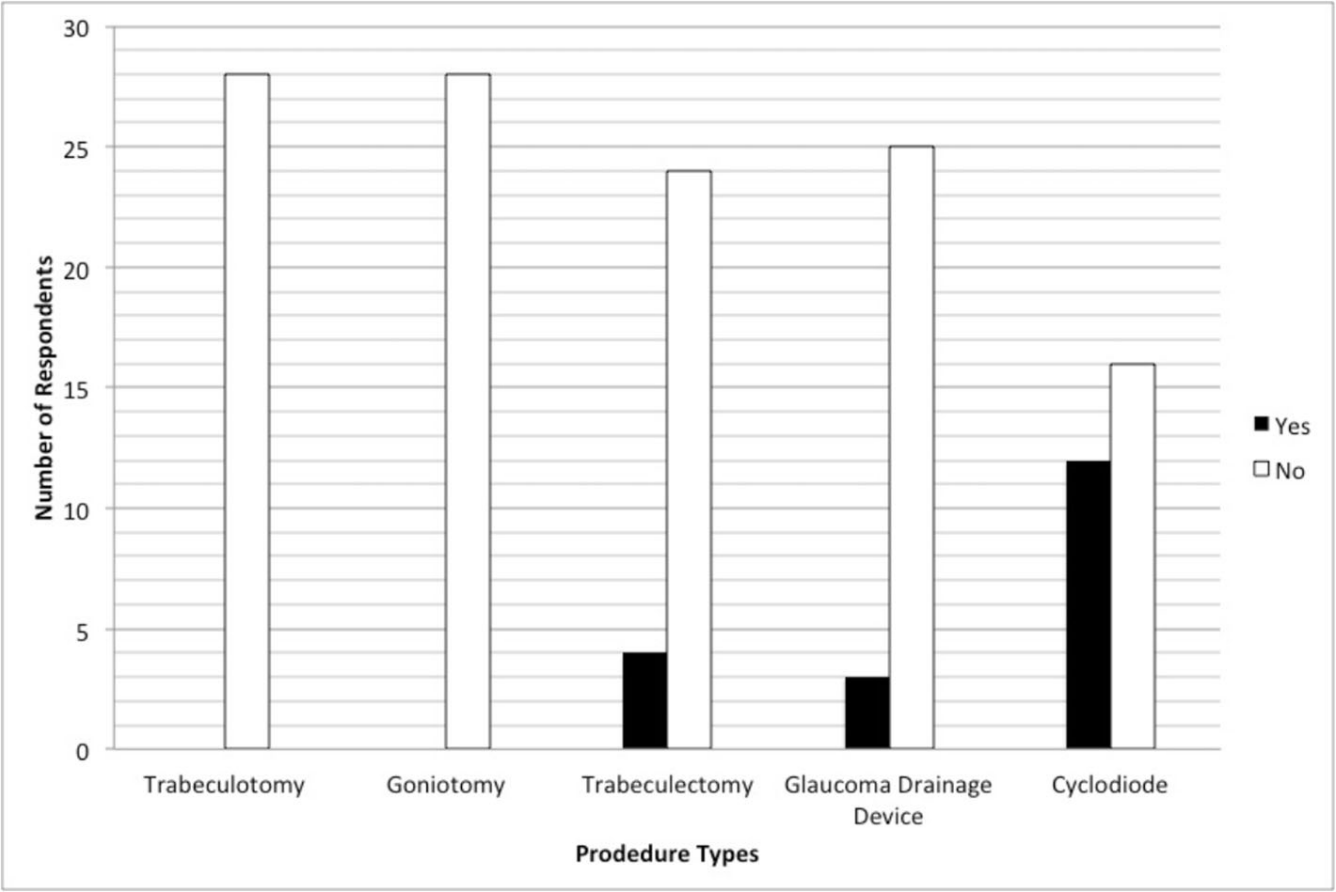
Responses to the question; ‘Do you feel comfortable performing the following surgical procedures on children?’ out of 28 respondants

**Fig. 3:**
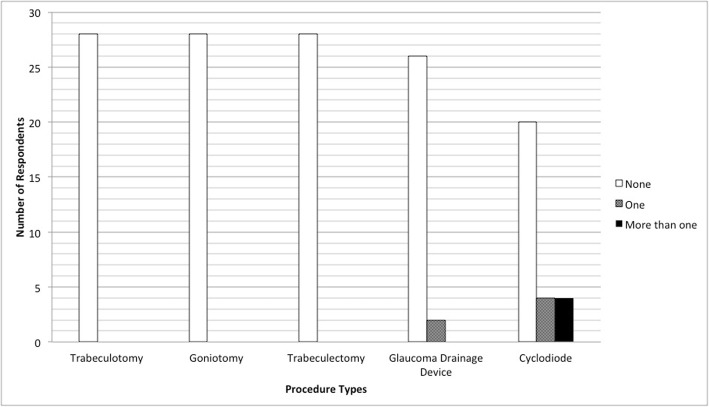
The number of procedures performed on children in the last 2 years

Regarding experience and comfort performing glaucoma surgery in children, no responders reported feeling comfortable performing goniotomy or trabeculotomy in children (Fig. 2). 4 (14.3%) responders reported they were comfortable performing trabeculectomy in children and 3 (10.7%) reported they were comfortable performing glaucoma drainage device surgery in children. 12 (42.9%) felt comfortable performing transscleral cyclodiode in children. Few procedures had been performed in children during the last 2 years, with no responders reporting that they had performed goniotomy, trabeculotomy or trabeculectomy on children in the last 2 years and only 2 consultants (both glaucoma sub-specialists) reporting one glaucoma drainage device procedure each. Five paediatric ophthalmologists and 3 glaucoma specialists had performed cyclodiode laser on a total of 14 children (Fig. 3). In total 11 children were referred to Moorfields Eye Hospital London for treatment for childhood glaucoma, 6 children were referred to Glasgow, 5 to Edinburgh, 1 to Manchester and 1 to Great Ormond Street.

## Discussion

Childhood glaucoma is rare and difficult to manage, with this survey identifying 85 new cases in Scotland over a 2-year period. The most common forms of glaucoma recognised were glaucoma associated with acquired conditions, particularly uveitis, and glaucoma following cataract surgery. Twenty-eight ophthalmologists responded to the survey, meaning on average consultants saw only 1.5 children with glaucoma per year. This is a very low number of patients, and likely below the number necessary to maintain skills in optimal management. It is important to emphasise that some responders saw considerably more patients and that our survey was aimed at determining the number of new diagnoses of glaucoma and not the overall exposure of consultants to children with a known glaucoma diagnosis. Nevertheless, perhaps due to the low number of children seen, few ophthalmologists responding to the survey reported feeling comfortable performing glaucoma surgery in children and few had performed any surgical glaucoma procedures for children in the previous 2 years. No responders were comfortable performing goniotomy or trabeculectomy, only 4 were comfortable performing trabeculectomy in children, and only 3 comfortable performing glaucoma drainage device surgery.

Surgery, when required, is normally performed in a high-volume tertiary centre by sub-specialist ophthalmologists. In keeping with a trend for subspecialisation within most paediatric surgical disciplines, by concentrating patients into a few centres it is likely a greater quality of care can be achieved. One of the principal downsides to this approach is the need for patients and their families to travel long distances on a regular basis, which can be costly and inconvenient as appointments are likely to clash with school and work commitments. Scotland has a relatively low population density compared to England and so it is important to consider the number of patients with childhood glaucoma and whether this would be enough to sustain a specialist unit. Eighty-five new cases of paediatric glaucoma of all subtypes were reported in Scotland over a two-year period by 28 ophthalmologists. Due to the response rate to the questionnaire, it is possible that this is an underestimation of the volume of childhood glaucoma in Scotland. Thirteen of these children required surgery that could only be performed in England. The 2011 census recorded 854,000 children under the age of 15 in Scotland. This would suggest an incidence of just over 5/100,000 new cases of childhood glaucoma in Scotland.

In comparison to the BIG Eye study published in 2007, a significantly higher proportion of secondary paediatric glaucoma was reported in Scotland. Seventy-four cases out of 85 (87%) were secondary glaucoma in comparison to 52 cases out of 99 (52.5%) as reported in the BIG Eye study [4]. However, the breakdown of the different subtypes of secondary glaucoma was similar. Lens-related and uveitic glaucoma were by far the most common (Table 1). The discrepancy in the proportion of secondary paediatric glaucoma between the BIG Eye study and our results could be due to the differences in population ethnicity between Scotland and the rest of the U.K., as only 4% of the Scottish population were from a minority ethnic group in the 2011 census, compared to 14% in England and Wales [7]. However, we did not record ethnicity in our survey and there is also the possibility that primary paediatric glaucoma cases were underestimated in our survey.

Comments from respondents further imply that the paucity of cases in individual ophthalmology units leads to a lack of experience and subsequently a lack of confidence by most clinicians. One respondent commented:

*‘Numbers are so low- challenge to keep up skills to provide highest quality care. Best left to high volume units to deal with this uncommon yet serious problem.’*

The final question of the survey examined the respondents’ feelings towards the development of a tertiary referral centre for paediatric glaucoma in Scotland. 85.7%(24/28) of respondents felt that Scotland needed a tertiary paediatric glaucoma service to manage these cases. In these cases, most respondents commented they felt Scotland should have a ‘centre of excellence’ for paediatric glaucoma.

*‘I feel that 1 centre should be able to offer a national paediatric glaucoma service including goniotomies and cyclodiode laser. Whether tubes can be done here depends on numbers of cases.’*

*‘These conditions are rare but devastating. We should move to one centre of excellence for Scotland.’*

*‘Even just a basic goniotomy/tubes service would probably be enough, I wouldn't mind sending really rare anterior segment cases or refractory cases to England. A single centre would be ideal in order to have sufficient case load.’*

Of the remaining 4 respondents, 2 (7.1%) felt that Scotland did not need a tertiary referral unit for paediatric glaucoma and 2 (7.1%) did not respond. Comments from these respondents expressed concern that there were insufficient patient numbers to justify a tertiary centre. One respondent stated they were happy with their current arrangement of referring paediatric glaucoma patients to services in England.

*‘My feeling is that the current arrangements in place for referring to London have been well received by patients' families; they have had a good service.’*

*‘Any arrangement in Scotland would have to be linked with a big centre in England. The whole population of Scotland is smaller than the big cities in England.’*

One respondent commented on some of the necessities in setting up a tertiary referral unit in Scotland and what would be required in order to provide a safe and holistic service.

*‘Paediatric ophthalmology colleagues are best placed to manage these patients given their close links with general paediatricians and the facilities to appropriately manage concurrent amblyopia and I would suggest a paediatric ophthalmologist with an interest in glaucoma rather than the other way round would be able to provide the best service. If a post(s) were to be established in Scotland, consideration would need to be given to having the proper support infrastructure including nursing expertise, parental support and flexible anaesthetic cover to manage these very challenging patients safely.’*

It is important to emphasise the limitations of the study including that the survey relied on clinician’s recalling numbers of patients seen and the results of the survey are limited by a response rate of only 56%. When considering the small numbers of children with glaucoma, it is possible that results may be significantly affected if one or two surgeons who saw large numbers of children with glaucoma failed to respond. To reduce the risk of this occurring we personally contacted surgeons already known to be seeing children with glaucoma to ensure that they had completed the survey.

## Conclusions

In conclusion, this survey reflects an appetite for paediatric glaucoma service in Scotland and undoubtedly such a service would be more convenient to patients and their families. However, further consideration is needed with regard to providing a service with a sufficient patient load to maintain the skills of an ophthalmologist to a level comparable to a tertiary centre in England.

## Data Availability

The datasets used and/or analysed during the current study are available from the corresponding author on reasonable request.

## Abbreviations

BIG Eye Study: The British Infantile and Childhood Glaucoma Eye Study
CGRN: Childhood Glaucoma Research Network
IOP: Intraocular Pressure
JOAG: Juvenile Open Angle Glaucoma
PCG: Primary Congenital Glaucoma
REDCap: Research Electronic Data Capture
